# Ostéoblastome du cornet moyen: tumeur rare du massif facial à ne pas méconnaitre

**DOI:** 10.11604/pamj.2019.32.24.13902

**Published:** 2019-01-16

**Authors:** Souha Kallel, Moncef Sellami

**Affiliations:** 1 Service ORL et Chirurgie Cervico-Faciale, CHU Habib Bourguiba, 3029 Sfax, Tunisie

**Keywords:** Ostéoblastome, massif facial, chirurgie, Osteoblastoma, facial massif, surgery

## Abstract

Osteoblastoma is a rare benign bone tumor, accounting for 1% of all bone tumors. It mainly occurs in the long bones, more rarely in the jaws. Osteoblastoma of the jaws mainly involves the mandible while nasosinusal involvement is very rare. The diameter of the tumor can grow to 10 cm. On X-ray, the tumor may appear as a well or poorly defined clear lesion, usually scattered with mineral patches. Recurrence rate is very low after surgical resection as well as the risk of malignant transformation. We report the case of a 13-year old female patient presenting with nasal obstruction evolving over the last 3 months associated with episodes of epistaxis and left blurred vision. Clinical examination showed voluminous nasal concha with polyploid mucosa filling all the left nasal fossa with left axial exophthalmos. CT scan of the facial massif showed left frontoethmoidal expansive process measuring 50*47*36 mm, spontaneously hypodense, heterogeneous, non enhanced after injection of the Contrast materials, responsible for destruction of the ethmoidal labyrinth, swelling of the bony walls with mass effect on the ipsilateral orbit. The suspected diagnosis was frontoethmoidal mucocele. Hence, the decision to perform endonasal surgery. Whitish thick liquid was collected by the cranial portion of the middle nasal concha sugesting infected mucocele. Resection of the middle nasal concha was performed. The histological examination showed osteoblastoma of the middle nasal concha . Outcome was marked by regression of the exophthalmos with no recurrence during 1-year follow up period.

## Image en médecine

L'ostéoblastome est une tumeur osseuse bénigne rare, représentant 1% de toutes les tumeurs de l'os. Elle touche essentiellement les os longs, plus rarement les mâchoires. Les atteintes au niveau des mâchoires sont surtout retrouvées à la mandibule, l'atteinte naso-sinusienne est très rare. Le diamètre de la tumeur peut atteindre 10cm. A la radiographie, la tumeur peut avoir l'aspect d'une lésion radio claire bien ou mal définie, généralement parsemée de plaques de minéralisation. Le taux de récidive est très faible après exérèse chirurgicale et le risque de transformation maligne est très faible. Nous rapportons le cas d'une patiente âgée de 13 ans qui a consulté pour une obstruction nasale évoluant depuis 3 mois avec des épisodes d'épistaxis et un flou visuel gauche. L'examen a trouvé un volumineux cornet à muqueuse polypoïde comblant toute la fosse nasale gauche avec une exophtalmie gauche axile. Le scanner du massif facial a montré un processus expansif fronto-ethmoïdal gauche de 50 x 47 x 36mm, spontanément hypodense hétérogène non réhaussé après injection du produit de contraste (PDC), responsable d'une destruction du labyrinthe ethmoïdal, d'une souflure des parois osseuses avec effet de masse sur l'orbite homolatéral. Le diagnostic évoqué était une mucocèle fronto-ethmoïdale. D'où la décision d'opérer par voie endonasale. La section de la tête du cornet moyen a ramené du liquide épais blanchâtre rappelant une mucocèle infectée. Nous avons complété par une résection du cornet moyen. L'examen histologique a conclu à un ostéoblastome du cornet moyen. Les suites étaient marquées par une régression de l'exophtalmie sans récidive après un recul de 1 an.

**Figure 1:**
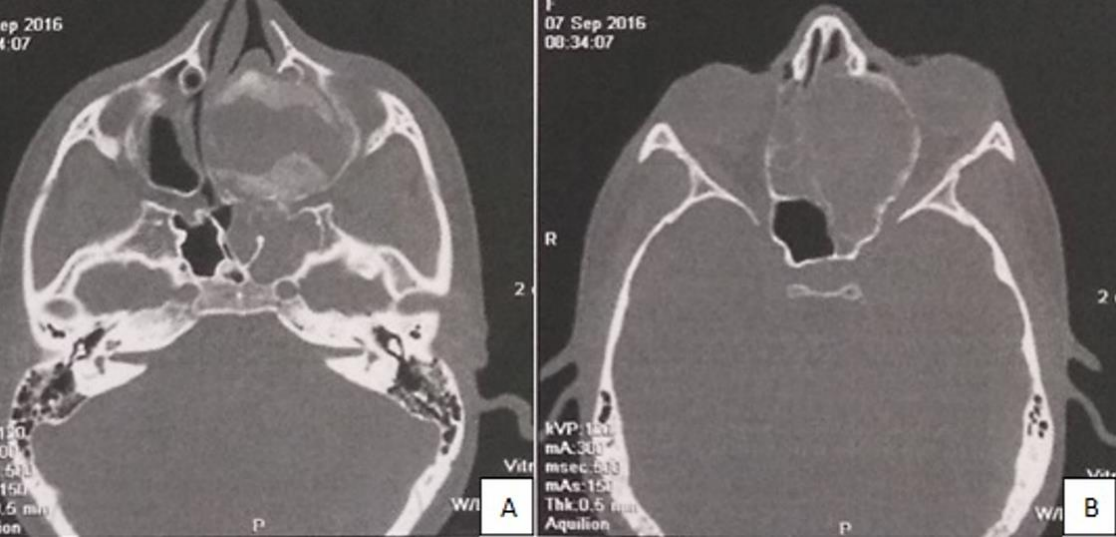
(A) processus expansif fronto-ethmoïdal gauche spontanément hypodense hétérogène non réhaussé après injection PDC responsable d'une destruction du labyrinthe ethmoïdal et d'une souflure des parois osseuses; (B) effet de masse en bas sur le cornet inférieur, en dehors sur l'orbite avec amincissement de la lame papyracée refoulant en dehors le muscle droit interne et responsable d'une exophtalmie grade I et d'une hypotrophie du nerf optique gauche

